# Organocatalytic activity of granaticin and its involvement in bactericidal function

**DOI:** 10.1038/s41598-022-10877-7

**Published:** 2022-04-29

**Authors:** Tatsuya Nishiyama, Narumi Enomoto, Reina Nagayasu, Kenji Ueda

**Affiliations:** Life Science Research Center, College of Bioresource Sciences, Nihon University, 1866 Kameino, Fujisawa, 252-0880 Japan

**Keywords:** Biochemistry, Microbiology, Chemistry

## Abstract

We previously discovered that actinorhodin, a benzoisochromanequinone antibiotic produced by *Streptomyces coelicolor* A3(2), serves as a catalyst facilitating the oxidation of ascorbic acid and cysteine (PNAS 48:17,152, 2014). In the present study, we screened for similar ascorbic acid-oxidizing activity in the culture broth of various *Streptomyces* spp., and discovered marked activity in the culture broth of *Streptomyces vietnamensis*. The principle active compound was granaticin, a pigmented antibiotic that is structurally related to actinorhodin. The absence of any metals in the purified granaticin fraction indicated that granaticin was an organocatalyst. Granaticin catalyzed the oxidation of L-ascorbic acid, generating L-dehydroascorbic acid and hydrogen peroxide (H_2_O_2_) at a 1:1 stoichiometric ratio, with 15 times higher reactivity than that of actinorhodin at an optimum pH of 7.0. Granaticin also oxidizes sulfhydryl compounds, including L-cysteine and glutathione. Growth inhibitory assays demonstrated that knockout mutants of the catalase gene exhibit high sensitivity to granaticin. The results suggest that the bactericidal activity of granaticin is exerted by the oxidation of sulfhydryl groups of cellular components and the toxicity of H_2_O_2_ generated during the oxidation reaction.

## Introduction

Microbial products have a vast structural and functional diversity. Since the discovery of penicillin and streptomycin, many compounds of microbial origin have played a significant role as therapeutic agents for various diseases^1^. Some compounds have also contributed to the progress of basic science as tools for studying the mechanistic details of biological functions^2,3^. The activities of these microbial products are mostly exerted through their association with a specific enzyme or other macromolecules, causing inhibition or disorder of the corresponding bioprocesses.

**Figure 1:**
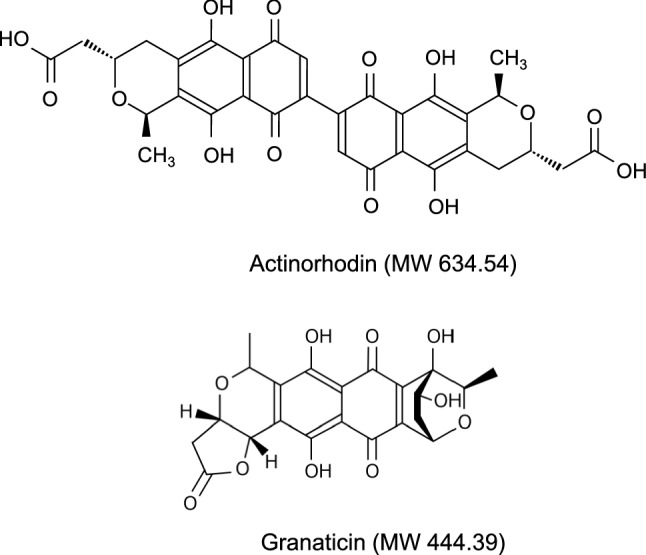
Chemical structure of actinorhodin and granaticin.

Previously, we discovered that actinorhodin (Fig. 1), a pigment antibiotic produced by *Streptomyces coelicolor* A3(2), retained its catalytic activity^4^. The addition of purified actinorhodin facilitated the oxidation of L-ascorbic acid and cysteine according to the following equations:

$${\text{L - ascorbic acid }} + {\text{ O}}_{{2}} + {\text{ H}}_{{2}} {\text{O}} \to {\text{L - dehydroascorbic acid }} + {\text{ H}}_{{2}} {\text{O}}_{{2}}$$

$${\text{L - cysteine }} + {\text{ O}}_{{2}} \to {\text{L - cystine }} + {\text{ H}}_{{2}} {\text{O}}_{{2}}$$

Because actinorhodin did not change its structure after these reactions, it was concluded that the promotion of the reaction was due to the catalytic function of actinorhodin.

Catalysts are grouped into types according to their physicochemical features and modes of action. Among these types, the major group is metal-containing catalysts, which are widely used in industrial chemical synthesis. However, the reactions carried out within living organisms are mostly mediated by biocatalysts. The major class of biocatalysts are enzymes, which are proteinaceous macromolecules, but some RNA molecules, termed ribozymes, also exhibit a catalytic function. Enzymes are utilized as an effective tool in industrial production because of their superior reactivity, including high stereoselectivity and low economic and environmental burdens.

The third group of biocatalysts is organocatalysts. Organocatalysts are catalytic organic molecules that do not contain metals^5^. The occurrence of catalytic activity in some organic compounds led to the hypothesis that the activity of some organic molecules that facilitates an asymmetrical chemical reaction is fundamental to the introduction and spread of homochirarity in the living creatures^6,7^. Although organic molecules were used as catalysts in the early chemical synthesis performed during the 1800s, their effective introduction to chemical synthesis began only after the year 2000^6,7^. Since then, the number of studies referring to the usefulness of organocatalysts especially in the enantio-selective synthesis has increased remarkably, reflecting their growing potential^7^. Proline and imidazolidinones are well-characterized organocatalysts^8–10^. In 2021, the Nobel Prize in Chemistry was awarded to Drs MacMillan and List for the development of asymmetric organocatalysts^11^. Our previous discovery of actinorhodin was the first instance of an organocatalyst produced by microbial secondary metabolism.

In the present study, we screened for additional *Streptomyces* metabolites exhibiting ascorbic acid-oxidizing activity to further explore the occurrence of organocatalysts in microbial metabolites. We discovered that granaticin, a benzoisochromanequinone antibiotic produced by *Streptomyces vietnamensis*, is catalytically active. This discovery not only highlights the use of microbial products as organocatalysts, which may be important in industrial applications, but also deepens the insights into the diversity of action mechanism of antibiotics and their role in the natural environment.

## Results

### Identification of granaticin as a catalytic compound

**Figure 2:**
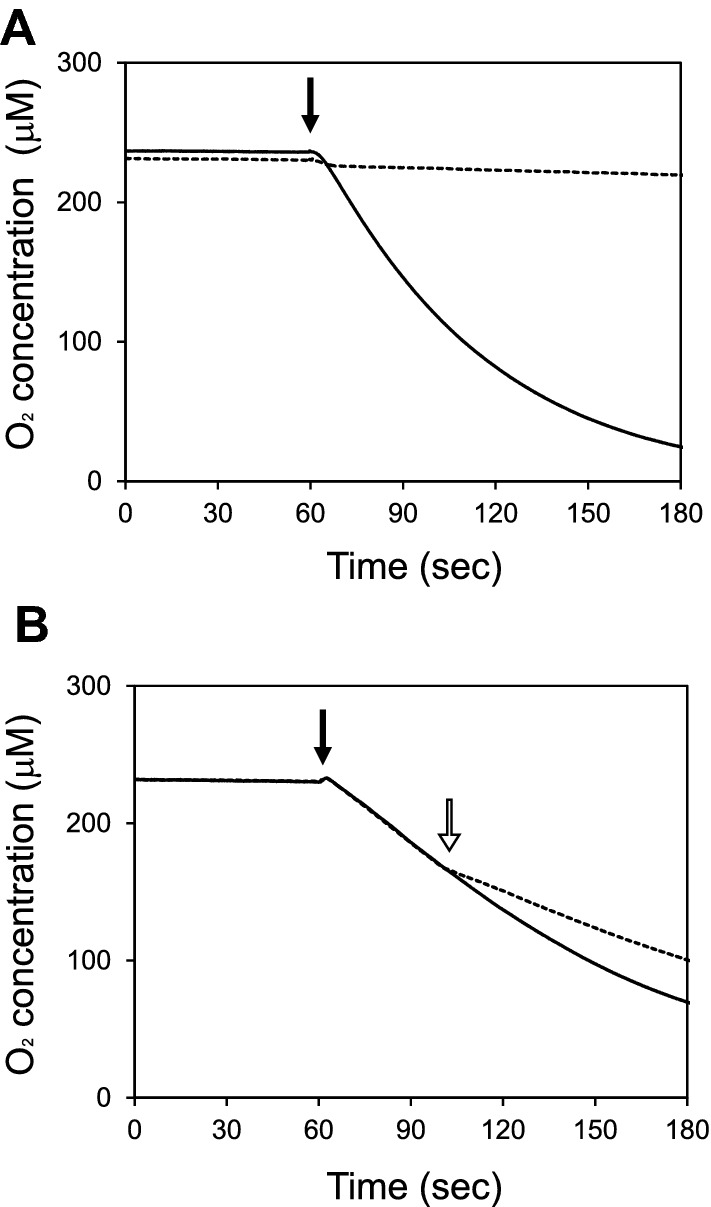
O_2_ consumption due to catalytic activity of granaticin. The dissolved O_2_ concentration in 10 mM L-ascorbic acid solution added to the culture supernatant of *S. vietnamensis* (**A**) and purified granaticin (0.03 mM) (**B**) was monitored using an oxygen electrode. The time points when the *S. vietnamensis* culture supernatant (**A**) and purified granaticin (**B**) were added are indicated by solid arrows. The open arrow in (**B**) indicates the time point for the addition of 100 units catalase. The dotted lines are the data from the measurement without the addition of culture supernatant (**A**) and with the addition of catalase (**B**).

To study the occurrence of organocatalytic activity in *Streptomyces* metabolites, we assessed the L-ascorbic acid-oxidizing activity in the culture supernatant of various *Streptomyces* strains (see “Methods”), and discovered that the culture broth of *S. vietnamensis* exhibited marked O_2_ consumption when it was mixed with 10 mM L-ascorbic acid (Fig. 2A). The same level of O_2_-consuming activity was observed even after heat treatment at 95 °C for 30 min (data not shown), indicating that the catalyst is a heat-stable molecule.

The culture broth of *S. vietnamensis* was purple in color, which was likely due to the production of a purple benzoisochromanequinone antibiotic, known as granaticin (Fig. 1)^12^. Previously, we proposed that the catalytic activity of actinorhodin is based on its quinone structure, since a weak but distinct L-ascorbic acid oxidizing activity was observed with several quinolic compounds of plant origin^4^. These previous observations led us to speculate granaticin-induced catalytic activity was present in the culture supernatant of *S. vietnamensis*. Therefore, the principle active compound was isolated using a previously published purification method for granaticin^13^. As expected, the L-ascorbic acid-oxidizing activity was fractionated into the same fraction as that of granaticin.

**Figure 3:**
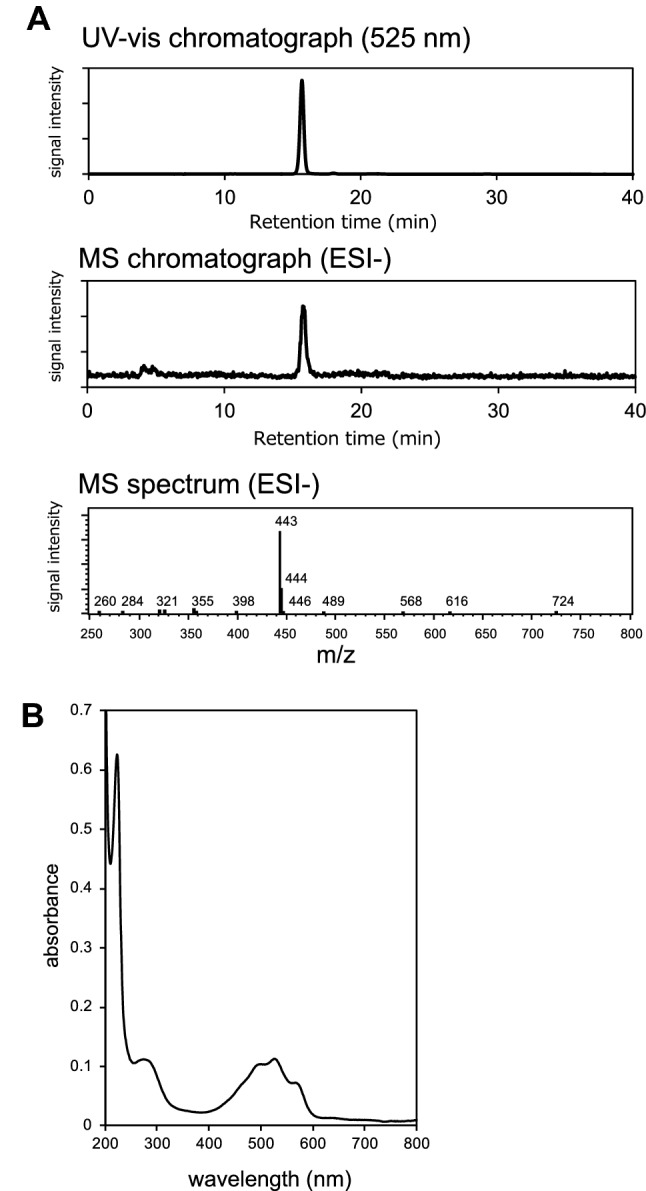
Spectra of the purified catalytic substance. LC–ESI–MS chromatogram (**A**) and UV–vis absorption spectrum (**B**) of the purified active compound. The single peak detected at 15.6 min (panel **A**, upper) was 443 m/*z* in the negative analytical mode according to MS analysis (panel **A**, lower), which coincided with the [M-H]^-^ *m/z* of granaticin. The UV–vis absorption spectrum of the purified fraction (panel **B**) containing peaks at 220, 285, 490, 525 and 570 nm was identical to the profile of purified granaticin reported by James and Edwards^14^.

LC–MS analysis of the resultant purified active fraction showed a single peak at 443 m/z (ESI, negative) (Fig. 3A). The UV–visible absorption spectrum of this fraction containing peaks at 220, 285, 490, 525 and 570 nm (Fig. 3B) was identical to that of granaticin previously reported by James and Edwards^14^. These results confirm that the isolated fraction contained purified granaticin. The metallic ion content of the fraction was determined using an inductively coupled plasma optical emission spectrometer (ICP-OES), but none of the 71 metallic ion species that could be identified by the analytical method were detected.

### Catalytic activity of granaticin

**Figure 4:**
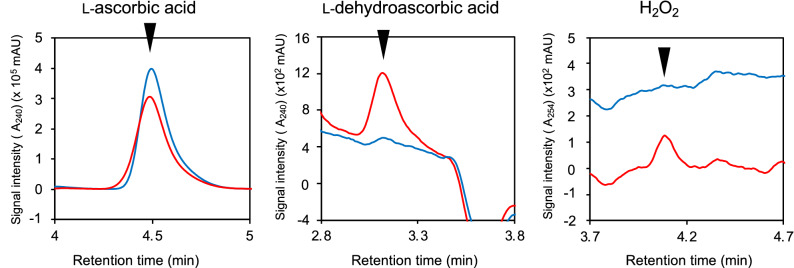
HPLC chromatogram of the reactants. The peaks corresponding to L-ascorbic acid, L-dehydroascorbic acid, and H_2_O_2_ before (blue line) and after (red line) the 5 min reaction with granaticin are shown.

**Figure 5:**
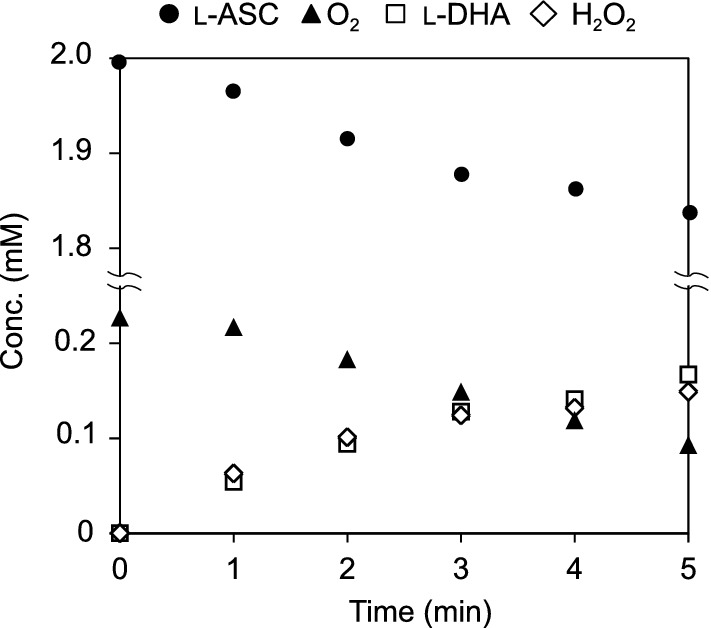
Stoichiometric analysis of granaticin catalysis. Time course of the concentration of L-ascorbic acid (L-ASC), O_2_, L-dehydroascorbic acid (L-DHA), and H_2_O_2_ in the oxidation reaction catalyzed by granaticin.

**Figure 6:**
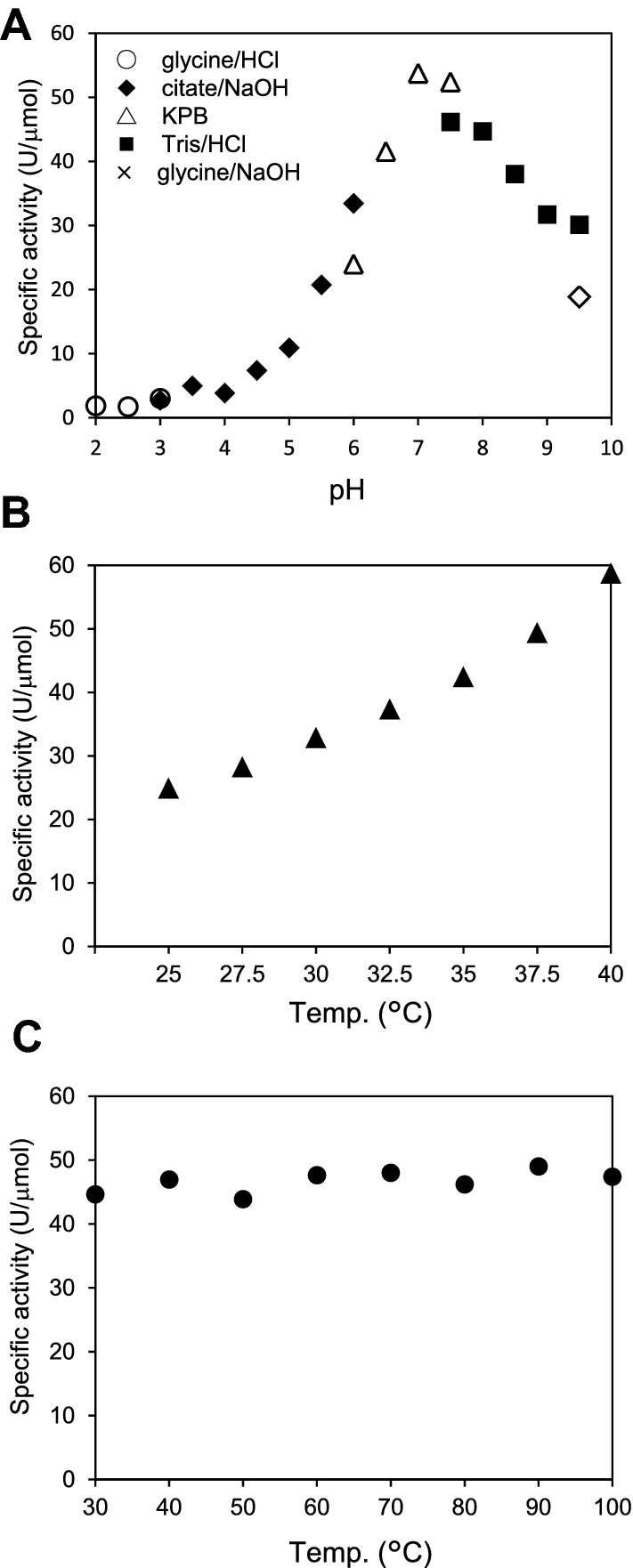
Effect of pH and temperature on granaticin activity. The catalytic activity of granaticin was measured at various pH values at 30 °C (**A**) and at various temperatures at pH 7.0 (**B**). The activity at temperatures higher than 40 °C could not be measured because of the operation limits of the oxygen electrode. The thermostability of the compound (**C**) was also studied by incubating granaticin solution at various temperatures for 1 h prior to the activity measurement. A representative result of repeated experiments is shown.

(i) **Specific activity**
  The L-ascorbic acid oxidizing activity of purified granaticin was determined. As shown in Fig. 2B, the dissolved O_2_ concentration in the reaction solution started to decrease immediately after mixing with granaticin. The addition of catalase reduced the ratio of O_2_ consumption (Fig. 2B dashed line), indicating the occurrence of H_2_O_2_ in the reaction mixture. LC–MS analysis of the reaction mixture revealed the presence of granaticin at the starting concentration, and the absence of any derivatives (Supporting information figure S1), indicating that granaticin catalyzes the oxidation of L-ascorbic acid without changing its structure or being destroyed.
  The specific activity of granaticin in L-ascorbic acid oxidation was 53.7 U/μmol, which is approximately 15-fold that of actinorhodin (3.5 U/μmol)^4^.
(ii) **Reaction products and stoichiometry**
  Our previous work demonstrated that actinorhodin catalyzes the oxidation of L-ascorbic acid, generating L-dehydroascorbic acid and H_2_O_2_^4^. To determine whether this was the case with granaticin, the content of the reaction mixture was analyzed by HPLC. The two peaks generated by the reaction with granaticin corresponded to those of L-dehydroascorbic acid and H_2_O_2_ (Fig. 4). This indicates that granaticin catalyzes the same reaction as actinorhodin.
  The stoichiometry of granaticin was determined (Fig. 5). The substrates (L-ascorbic acid and O_2_) and products (L-dehydroascorbic acid and H_2_O_2_) were present at a 1:1:1:1 stoichiometric ratio.
(iii) **Dependence on pH and temperature**
  The pH and temperature dependence of the catalytic activity of granaticin was assessed. The highest activity was observed at pH 7.0 (Fig. 6A). The oxidation of L-ascorbic acid occurred spontaneously at pH 10 and higher, as described previously^4^. The activity increased with increasing temperature from 25 to 40 °C (Fig. 6B). The activity at temperatures higher than 40 °C could not be measured because of the heat sensitivity of the electrode system. Although data were slightly fluctuated, preincubation at temperatures between 30 and 100 °C for 1 h did not significantly affect the catalytic activity (Fig. 6C).
(iv) **Substrate specificity**
  The substrate specificity of granaticin was determined by reacting granaticin with various substrates. The substrate selectivity profile was similar to that of actinorhodin^4^, but the reactivity was different (Table 1). Compared to L-ascorbic acid (100%), the reaction of its stereoisomer D-isoascorbic acid was relatively weak (77.3%), indicating that the catalysis occurs asymmetrically. Much weaker but distinctive reactions were observed with L-cysteine methyl ester (1.5%), L-cysteine (0.6%), dithiothreitol (0.4%), D-cysteine (0.4%), and glutathione (0.1%). The narrow but distinctive difference in the reactivity with cysteine isomers also indicates the asymmetricity of the reaction. Oxidation of *N*-acetyl-L-cysteine, DL-penicillamine, DL-homocysteine, lipoamide, and α-lipoic acid was not observed when O_2_ consumption was monitored for 30 min.

**Table 1:** Substrate specificity.

Substrates	Catalytic activity (U/μmol)	
	Granaticin	Actinorhodin
L-ascorbic acid	52.63	3.50
D*-*iso-ascorbic acid	40.66	3.01
L-cysteine methyl ester	0.77	0.56
L-cysteine	0.31	0.02
Dithiothreitol	0.22	0.34
D-cysteine	0.19	NT
Glutathione	0.06	0.01
*N*-acetyl-L-cysteine	ND	ND
DL-penicillamine	ND	ND
DL-homocysteine	ND	ND
Lipoamide	ND	NT
α-lipoic acid	ND	NT

*ND* not detected, *NT* not tested.

### Growth inhibition activity of granaticin

We previously hypothesized that the antibiotic activity of actinorhodin is at least partly due to the toxicity of H_2_O_2_ generated along with the catalytic reaction, based on the observation that the addition of catalase rescued the growth of *S. aureus* exposed to actinorhodin on solid media^4^. The same assay using the filter disc diffusion method showed that the growth inhibition effect of granaticin on *S. aureus* was also ameliorated by the addition of catalase (data not shown).

**Figure 7:**
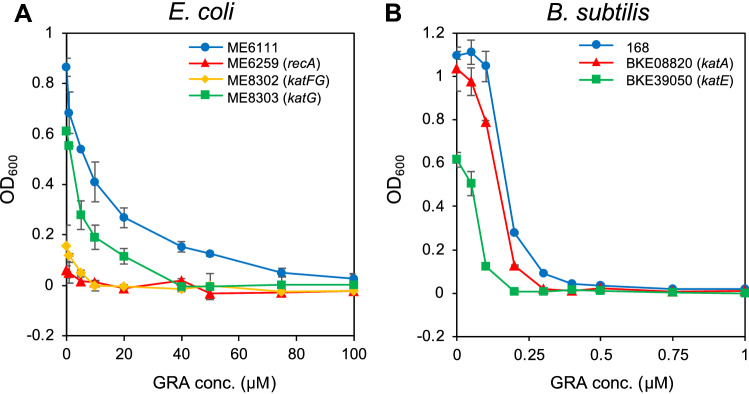
Inhibition of bacterial growth by granaticin. Growth inhibition of *E. coli* (**A**) and *B. subtilis* (**B**). Strains were inoculated into LB liquid medium containing various concentrations of granaticin, and the optical density at 600 nm (OD600) was measured after 5 h of cultivation. The data are the means of three measurements. The error bars represent the standard deviation.

We next studied the sensitivity of catalase-deficient strains of *E. coli* and *B. subtilis* (Table 2). Figure 7 shows the dose effect of granaticin on the early growth (5 h cultivation) of these mutants in Luria–Bertani (LB) liquid medium. Granaticin is known to be an antibiotic that statically inhibits the growth of gram-positive bacteria^15^. As expected, the sensitivity of *E. coli* was far lower than that of *B. subtilis* (ca, 100-foled difference). However, in both bacteria, the catalase-deficient mutants exhibited higher sensitivity to granaticin than the control strain. In *E. coli*, ME8302 lacking the two catalase genes (*katF* and *katG*) was more sensitive than ME8303, lacking active *katG* but retaining *katF* (Fig. 6A). We also performed the assay with an *E. coli* mutant (ME6259) for *recA*, the master regulator of the SOS response system^16^, and found that it had a higher sensitivity than the control strain (ME6111) (Fig. 6A). A similar profile was obtained for JM109 (*recA*-) and JM105 (*recA* +) strains (data not shown). Similarly, the mutant for *katA* (BKE08820) and *katE* (BKE39050) of *B. subtilis* exhibited higher sensitivities than the control strain (Fig. 6B). These results support our hypothesis that the toxicity of granaticin is based on the effect of H_2_O_2_ that is generated by its catalytic reaction. The high sensitivity of *recA* mutants could be due to their disability to repair the damage caused in DNA structure by the oxidative agent.

**Table 2:** Genotype of *E. coli* and *B. subtilis* mutants used in this study.

Strain	Genotype
***E. coli***	
ME6111	*asn thi thyA str*
ME6259	*asn thi thy str recA*
ME8302	*leuB6 proC83 purE42 trpE28 his-208 argG77 ilvA681 met-160 thi-1 ara-14 *l*acY1 galK2* *xyl-5 mtl-1 azi-6 rpsL109 tonA23 tsx-67 supE44 malA38 xthA katF3 kat-3 cysI::Tn10*
ME8303	*leuB6 proC83 purE42 trpE38 his-208 argG77 ilvA681 metA160 thi-1 ara-14lacY1 galK2* *xyl-5 mtl-1 azi-6 rpsL109 tonA23 tsx-67 supE44 malA38 xthA katG17::Tn10*
JM105	*endA hsdR*(rk-mk +) *rpsL sbcBC thi-1* Δ(*lac*-*proAB*) F'[t*raD36 proAB lacIqZ*ΔM15]
JM109	*recAl supE44 endA1 hsdR17 gryA96 thi* Δ(*lac*-*proAB*) F'[*traD36 proAB lacIqZ*ΔM15]
***B. subtilis***	
BKE08820	*katA*
BKE39050	*katE*

## Discussion

In the present study, we determined that granaticin serves as a catalyst to facilitate the oxidation of L-ascorbic acid to L-dehydroascorbic acid. The lack of structural changes or depletion during this reaction, as well as the lack of metallic content, indicate that granaticin is an organocatalyst. The relatively narrow but distinctive difference in the reactivity with the stereoisomers of ascorbic acid and cysteine (Table 1) indicates that the catalytic reaction occurs asymmetrically. This finding reinforces the notion that these types of functional molecules are present in microbial products.

**Figure 8:**
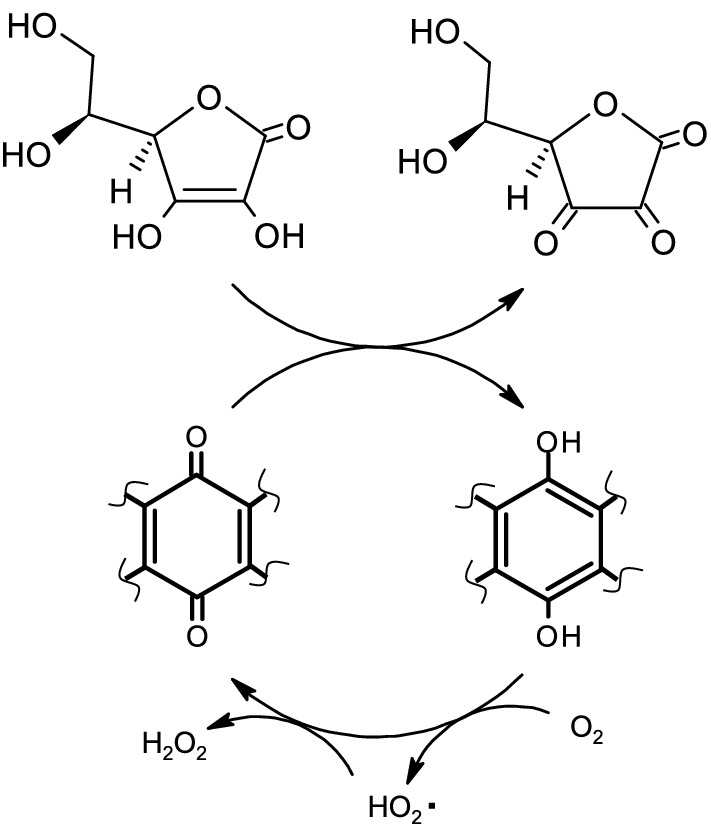
Hypothetical mechanism of catalysis. Possible mechanism of ascorbic acid oxidation catalyzed by granaticin and other quinolic substances. The catalytic reaction occurs because of the autonomous regeneration of the quinone form, via electron transfer from the quinol form to molecular oxygen.

Granaticin and actinorhodin belong to the same class of compounds, termed benzoisochromanequinones, which share a naphthoquinone structure (Fig. 1). We previously showed that some plant-derived natural products, which commonly retain a quinone structure, also exhibit catalytic activity, facilitating the oxidation of ascorbic acid^4^. Thus, we speculate that the quinone structure is fundamental to the catalytic function of this class of organocatalysts. We hypothesize that the structural interconversion between quinone and quinol within the catalytic molecule mediates the oxidation of ascorbic acid to dehydroascorbic acid and the reduction of O_2_ to H_2_O_2_ (Fig. 8). Granaticin differs from actinorhodin in terms of its specific activity, optimum pH, and substrate specificity. It is likely that the differences in the structure surrounding the quinone backbone affect the catalytic properties of these molecules. A detailed understanding of the correlation of their chemical structure with the reactivity will broaden the field of organocatalysts.

Granaticin was first identified in the culture broth of *Streptomyces olivaceus* based on its antibiotic activity against gram-positive bacteria^17^. It was also cytotoxic to mammalian cells. Chang et al.^18^ discovered that an antibiotic termed litmomycin, which is produced by *Streptomyces litmogenes*, exhibited antitumor activity against leukemia in mice, and that the chemical structure of litmomycin was identical to that of granaticin. Early studies on the bactericidal mechanisms of granaticin suggest that its interaction with RNA or related molecules is fundamental to its antibiotic activity. Ogilvie et al.^15^ studied the effects of granaticin on *Bacillus subtilis* and concluded that it interferes with the aminoacylation of leucyl transfer RNA, thereby inhibiting translation. However, other studies have proposed different targets of inhibition, such as RNA synthesis by RNA polymerase^15^, DNA synthesis by reverse transcriptase^19^, and maturation of ribosomal RNA^20^.

Independently of its possible interaction with RNA or related molecules, Sturdik and Drobnica^21^ determined that granaticin is reactive to sulfhydryl groups by observing its effect on sulfhydryl compounds and sulfhydryl enzymes, including glyceraldehyde-3-phosphate dehydrogenase, and proposed that this kind of activity is fundamental to the cytotoxicity of granaticin. This is consistent with the report by Gibson-Clay et al.^22^, who observed the inhibitory effect of granaticin on the activity of pyruvate decarboxylase, the central metabolic enzyme, depending on its activity on the oxidation and reduction of the SH group of lipoamide, which is associated with the E2 domain.

The ambiguity of the action mechanism of granaticin can be explained by its organocatalytic activity. Previously, we hypothesized that the bactericidal activity of actinorhodin is, at least in part, due to the H_2_O_2_ produced during catalysis, based on the observation that the addition of catalase reduced the level of growth inhibition^4^. H_2_O_2_ generated via the catalytic reaction exerts toxic effects on cellular functions. This is also supported by the antibiotic activity measurements performed in the present study (Fig. 7). The high sensitivity of the catalase mutants to granaticin suggests that the generation of H_2_O_2_ within or on the surface of the target cell, along with the oxidation of cellular material, is fundamental to the antibiotic and cytotoxic activity of this compound. The similar feature of the *recA* mutants is interpreted as the oxidative effect of H_2_O_2_ damages the chromosomal DNA and therefore significantly affects the viability of the *recA* mutant strains, which lack the ability to express the DNA repair system^16^. The oxidative damage to nucleic acid structures may be correlated with the abovementioned hypothesis that granaticin exerts its cytotoxicity by introducing damage to RNA and related functions.

Recently, Nass et al.^23^ reported that γ-actinorhodin, an actinorhodin derivative, exhibits unique antibacterial activity. The bactericidal activity of γ-actinorhodin was supposed to be due to the generation of reactive oxygen species (ROS), as the *S. aureus* mutant for superoxide dismutase was highly sensitive to the substance. Similar observations have been reported for the bactericidal activity of a plant-derived bioactive quinone termed thymoquinone^24^, a chemically synthesized anticancer and antibacterial compound YM155 with a naphthoimidazole structure^25^, and 1,4-naphtoquinones exhibiting antibacterial activity and cytotoxicity^26^. These compounds may also exert their toxicity by generating ROS. The mode of action of these other bioactive quinolic substances reinforces the view that the toxicity of granaticin is also based on the generation of ROS.

In addition to ROS generation, inactivation of proteins due to the oxidation of sulfhydryl groups could be the basis of the observed toxicity of granaticin and related compounds. Although the specific activity was relatively low, in the present study we determined that granaticin promotes the oxidation of cysteine. This suggests that the sulfhydryl group, which is exposed to the protein surface, could be the target of oxidation by granaticin. This agrees with the abovementioned observation that granaticin inhibits sulfhydryl enzymes. Overall, the lines of evidence strongly suggest the existence of a novel mechanism for the action of antibiotics and bioactive substances based on their catalytic activity.

The reactivity of granaticin at neutral pH and moderate temperature indicates that its catalytic function can proceed under standard conditions. The granaticin biosynthetic gene cluster exists in the genome of various *Streptomyces* and other actinomycetes^27,28^. Deng et al.^29^ suggested that the ability to produce granaticin is distributed via horizontal gene transfer. Thus, the newly discovered function of this natural product is widespread in the natural environment, and it is readily available for various oxidative reactions. Taken together, these features with regard to granaticin support the occurrence of an unprecedent role, function, and utilization of microbial products.

## Methods

### Bacterial strains and media

The *Streptomyces* strains used for catalytic activity screening were obtained from the National Institute of Technology and Evaluation (NITE), Chiba, Japan (Table 3). *Staphylococcus aureus* NBRC100910 was used for the growth inhibition assay, and was also obtained from the NITE. *Escherichia coli* strains used for the growth inhibition assay (Table 2) were obtained from the National Bioresource Project (NBRP) (https://nbrp.jp/en/). JM105 and JM109 were purchased from Takara Bio Inc. (Kyoto, Japan). *Bacillus subtilis* strains 168, BKE08820 (*katA*-deficient), and BKE39050 (*katE*-deficient) were also obtained from the NBRP.

**Table 3:** *Streptomyces* strains used for screening.

Species	NBRC no	Species	NBRC no
*S. abikoensis*	13907	*S. ahygroscopicus*	13664
*S. albofaciens*	12833	*S. albulus*	13410
*S. amakusaensis*	12835	*S. argenteolus*	12841
*S. argenteolus*	13847	*S. cellulosae*	13027
*S. chattanoogensis*	12754	*S. fasiculatus*	12765
*S. griseolus*	12777	*S. hygroscopicus*	3977
*S. hygroscopicus*	13815	*S. inusitatus*	13601
*S. libani*	13452	*S. melanogenes*	12890
*S. noboritoensis*	13065	*S. olivaceiscleroticus*	13484
*S. olivoverticillatus*	13068	*S. sanglieri*	100784
*S. setonii*	13085	*S. sioyaensis*	12820
*S. somaliensis*	12916	*S. sporoclivatus*	100767
*S. verne*	13097	*S. vietnamensis*	104153
*S. violens*	13486		

*Streptomyces* strains were cultured at 28 °C in Bennett’s/maltose (BM) medium containing 1 g/L yeast extract (Difco Laboratories, Detroit, Michigan), 1 g/L meat extract (Kyokuto, Tokyo, Japan), 2 g/L NZ amine (Wako Pure Chemical Industries Ltd., Osaka, Japan), and 10 g/L maltose (pH 7.2). For the antibiotic assay, *E. coli* and *B. subtilis* strains were cultured in Luria–Bertani (LB) medium containing 10 g/L tryptone (Difco), 5 g/L yeast extract (Difco), and 5 g/L NaCl. Agar was added to LB broth to a final concentration of 1.5% to prepare solid media. Chemicals were purchased from Kokusan (Tokyo, Japan), unless otherwise indicated.

### Screening for catalytic activity

Screening for the organocatalytic activity in *Streptomyces* culture supernatants was performed by monitoring the decrease in dissolved O_2_ concentration using an oxygen electrode (Hansatech Instruments Ltd., Norfolk, UK). Each *Streptomyces* strain was cultured in a test tube (Φ18 × 180 mm) containing 10 ml BM liquid medium at 28 °C for 5 days with reciprocal shaking at 300 rpm. After growth, the supernatant was obtained by centrifugation at 10,400×*g* at 4 °C for 15 min. The substrate solution contained 10 mM L-ascorbic acid and 100 mM potassium phosphate buffer (pH 7.2). The reaction was initiated by adding 20 µl of culture supernatant to 1 ml substrate solution in an electrode cuvette and incubating at 30 °C for 5 min. The consumption of O_2_ during the 5 min reaction was monitored using an oxygen electrode according to the manufacturer’s instructions. To evaluate the O_2_ decrease due to spontaneous oxidation, the decrease in O_2_ concentration was measured in parallel in a negative control mixture with fresh culture medium in place of the culture supernatant.

### Isolation of granaticin

*S. vietnamensis* NBRC 104153 was first inoculated into a test tube (Φ18 × 180 mm) containing 10 ml BM liquid medium and cultured at 28 °C for 5 days with reciprocal shaking at 300 rpm. Then, 1 ml of this preculture was inoculated into a baffled 500 ml Erlenmeyer flask containing 100 ml of BM medium. The culture was incubated at 28 °C with rotary shaking at 160 rpm for 1 day. The culture supernatant was obtained by centrifugation at 10,400×*g* at 4 °C for 15 min. The supernatant was added to an equivalent volume of ethyl acetate/methanol/acetic acid (80/20/5) to extract the active compounds. The organic phase was collected, evaporated to dryness, and dissolved in acetonitrile, and then fractionated using a Shimadzu LC-10Avp high-performance liquid chromatography (HPLC) system equipped with a COSMOSIL 5C18-MS-II column (4.6 mm i.d. × 150 mm; Nacalai Tesque, Inc., Kyoto, Japan) under the following conditions: column temperature, 40 °C; flow rate, 1 ml/min; photodiode array detector, 190–800 nm; mobile-phase solvent, 55% methanol; and isocratic elution for 15 min. The eluate corresponding to the isolated peak at 10.6 min was obtained and evaporated to dryness using a rotary evaporator. The dried material was then dissolved in methanol: water at a ratio of 55:45 due to its high solubility and stability, and was henceforth termed ‘granaticin solution’. The solution was maintained at 4 °C to avoid evaporation of methanol.

The molecular mass was determined by liquid chromatography electrospray ionization mass spectrometry (LC–ESI–MS; negative mode) with a Shimadzu LCMS-2010 system equipped with a COSMOSIL 5C_18_-PAQ column (4.6 mm i.d. × 150 mm; Nacalai Tesque) under the following conditions: column temperature, 40 °C; flow rate, 1 ml/min; and photodiode array detector, 190–800 nm; mobile phase, 0.5% (v/v) acetic acid in 50% methanol; isocratic elution for 40 min.

### Characterization of the catalytic activity of granaticin

The catalytic activity of granaticin was studied using an oxygen electrode under the same conditions as those used for activity screening. The dissolved O_2_ concentration in a 1 ml reaction mixture containing 20 µl of 1 µM granaticin solution was monitored for 5 min using an oxygen electrode. One hundred units of catalase (Nacalai Tesque, Inc., Kyoto, Japan) were added if needed. For the negative control measurement, 20 µl of 55% methanol was added in place of the granaticin solution to confirm that the presence of methanol does not affect the accurate measurement. The O_2_ consumption was also measured using a non-contact O_2_ sensor chip system (OXY-1 SMA; PreSensPrecision Sensing GmbH, Regensburg, Germany) for reactions where oxidation occurred slowly. The reaction mixture was prepared under the same conditions and enclosed in a glass vial containing a sensor chip attached to its inner surface. The dissolved O_2_ concentration was recorded every 1 min for up to 30 min by scanning the chip with the chip reader from the outside of the vial. One unit of catalytic activity was defined as the amount of the compound that catalyzed the consumption of 1 µmol of O_2_ per min.

To identify the reaction products generated by the catalytic oxidation of L-ascorbic acid, the reaction mixture was incubated at 30 °C for 5 min, and formic acid was added to a final concentration of 1% to stop the reaction. The reactant was then analyzed by HPLC (Shimadzu LC-10Avp system) on a ZIC-HILIC column (4.6 mm i.d. × 150 mm; Merck KGaA, Darmstadt, Germany) operated under the following conditions: column temperature, 40 °C; isocratic elution of 100 mM ammonium acetate:CH_3_CN = 3:7 mobile phase); flow rate, 1 ml/min; and photo-diode array detector, 240 nm. H_2_O_2_ was detected by HPLC on a COSMOSIL Sugar-D column (4.6 mm i.d. × 250 mm; Nacalai Tesque, Inc.) under the following conditions: column temperature, 40 °C; isocratic elution of 80% acetonitrile mobile phase; flow rate, 1 ml/min; and photodiode array detector, 254 nm.

The temperature dependence of the catalytic activity of granaticin was determined by measuring the reduction in O_2_ in the reaction mixture incubated at various temperatures between 25 and 40 °C. The composition of the reaction mixture was the same as that above, except for the addition of 2 mM L-ascorbic acid. pH dependence was analyzed by adding 2 mM L-ascorbic acid to 100 mM of the following buffers: glycine/HCl buffer (pH 2.0–3.0), citate/NaOH buffer (pH 3.0–6.0), potassium phosphate buffer (pH 6.0–7.5), Tris–HCl buffer (pH 7.5–9.5), and glycine/NaOH buffer (pH 9.5). Heat tolerance was investigated by incubating the granaticin solution at various temperatures for 1 h prior to activity measurement using the oxygen electrode as before. The reaction mixture conditions were the same as those used for the temperature dependence analysis.

### Metal analysis

All glassware was soaked in 0.3 M HNO_3_ overnight, and exhaustively rinsed with distilled water before use. Granaticin solution (20 μM) and 55% methanol (reference) were analyzed using an inductivity coupled plasma optical emission spectrometer (model ICPE-9000, Shimadzu, Kyoto, Japan). The analysis enabled the detection of the following 71 metals at concentrations > 5 ppb: lithium, beryllium, boron, sodium, magnesium, aluminum, silicon, phosphorus, sulfur, potassium, calcium, scandium, titanium, vanadium, chromium, manganese, iron, cobalt, nickel, copper, zinc, gallium, germanium, arsenic, selenium, rubidium, strontium, yttrium, zirconium, niobium, molybdenum, ruthenium, rhodium, palladium, silver, cadmium, indium, tin, antimony, tellurium, iodine, cesium, barium, hafnium, tantalum, tungsten, rhenium, osmium, iridium, platinum, gold, mercury, thallium, lead, bismuth, lanthanum, cerium, praseodymium, neodymium, samarium, europium, gadolinium, terbium, dysprosium, holmium, erbium, thulium, ytterbium, lutetium, thorium and uranium.

### Growth inhibition analysis

*E. coli* and *B. subtilis* strains were precultured for 16 h with shaking at 135 rpm in glass tubes containing 5 ml LB liquid medium at 37 °C and 30 °C, respectively, and then inoculated to a final concentration of 0.5% into the culture medium under the same conditions as the preculture, to which granaticin was added at 0–100 μM (*E. coli*) and 0–1.0 μM (*B. subtilis*). After 5 h of cultivation, growth was quantified by measuring the optical density at 600 nm.

## Supplementary Information

Supplementary Information.

## Data Availability

All data are contained within the manuscript.
