# Supplementary material for: Organocatalytic activity of granaticin and its involvement in bactericidal function
Source: Sci Rep. 2022 Apr 29;12:7046. doi: 10.1038/s41598-022-10877-7 (PMC9054759; doi:10.1038/s41598-022-10877-7)
Supplement: Supplementary file 1 — Supplementary Information. [file 41598_2022_10877_MOESM1_ESM.pdf]

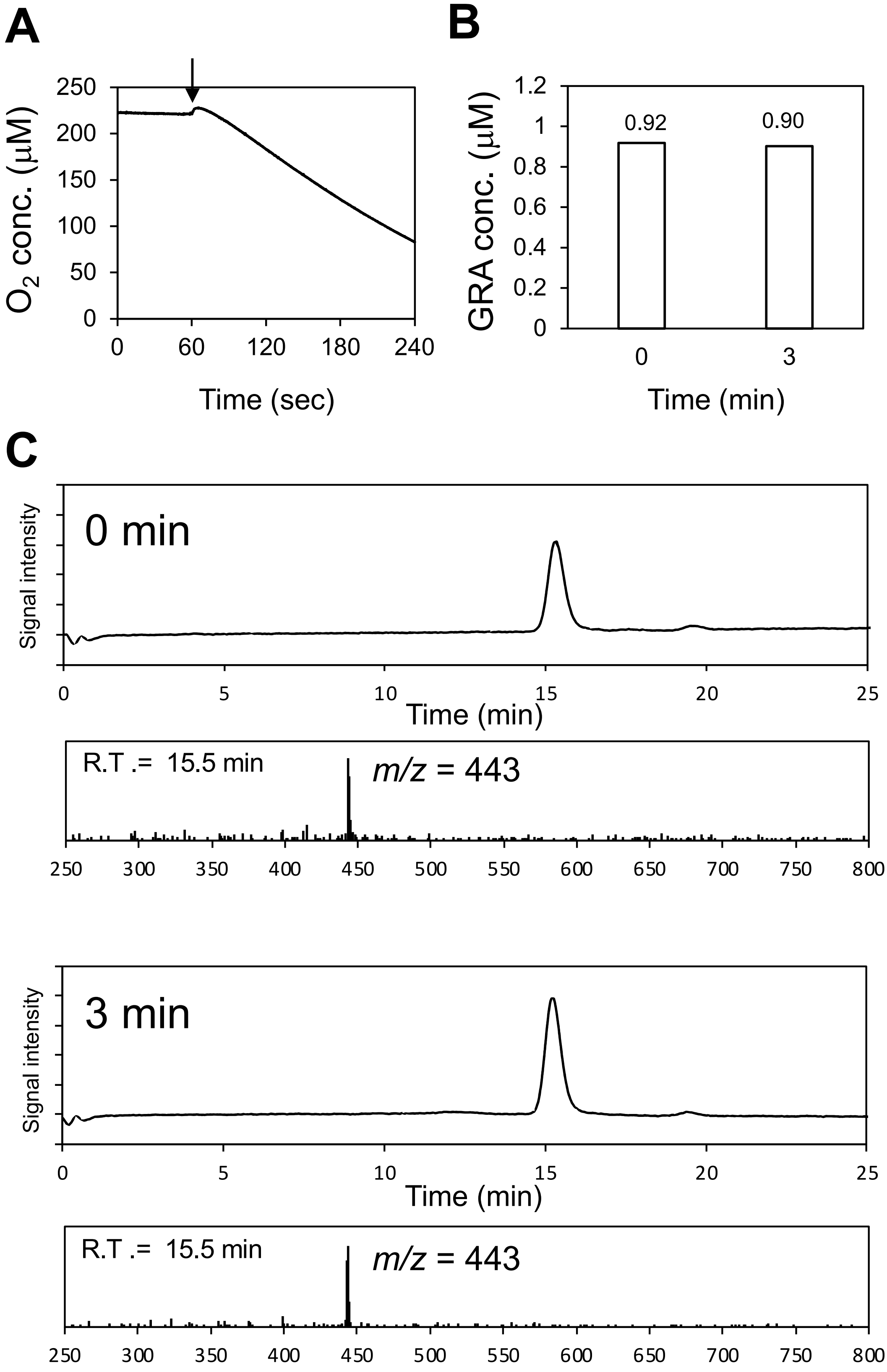

Supplementary figure S1. Unchangeability of granaticin after catalytic reaction. (A) O_2 consumption due to the catalytic activity of granaticin. The time point when purified granaticin was added is indicated by an solid arrow. (B) Concentration of granaticin in the reaction mixture before (left) and after 3 min reaction. (C) LC-ESI-MS chromatogram of the reaction mixture before (upper) and after (lower) 3 min reaction. Conditions are the same as the data presented in Fig. 2B.
